# Protein disulphide isomerase protects against ALS/FTD phenotypes associated with cyclin F in zebrafish and cellular models

**DOI:** 10.1080/13510002.2026.2732775

**Published:** 2026-10-18

**Authors:** Sina Shadfar, Sonam Parakh, Emma Perri, Cyril Jones Jagaraj, Sara Assar Kashani, Keelin Beyers, Audrey M.G. Ragagnin, Angela S. Laird, Julie D. Atkin

**Affiliations:** a Motor Neuron Disease Research Centre, Macquarie Medical School, Faculty of Medicine, Health and Human Sciences, Macquarie University, Sydney, NSW, Australia

**Keywords:** MND, FTD, neurodegeneration, proteostasis, ER stress, ageing, cyclin F, zebrafish, *CCNF*

## Abstract

**Background:**

Mutations in *CCNF*, encoding cyclin F, cause a small proportion of amyotrophic lateral sclerosis (ALS) and frontotemporal dementia (FTD) cases. Mutant cyclin F mislocalises from the nucleus to the cytoplasm, impairs the ubiquitin proteasome system, disrupts endoplasmic reticulum (ER) to Golgi transport, and triggers ER stress and apoptosis. Protein disulphide isomerase (PDI), a redox-active ER chaperone of the thioredoxin superfamily, protects against ALS phenotypes induced by mutant SOD1, TDP-43, and FUS, but its activity against mutant cyclin F has not been examined.

**Objectives:**

To test whether PDI overexpression prevents the cellular and behavioural phenotypes induced by the ALS/FTD-associated cyclin F^S621G^ mutation.

**Methods:**

Neuro-2a cells were co-transfected with mCherry-tagged wild-type or mutant cyclin F^S621G^ and V5-tagged PDI or empty vector. Subcellular localisation, XBP-1 and CHOP activation, proteasome reporter (GFPu) accumulation, nuclear morphology, and cleaved caspase-3 immunoreactivity were assessed by confocal microscopy. Transient transgenic zebrafish larvae generated by mRNA co-injection of cyclin F and PDI were assessed by photomotor response at 48 h post-fertilisation.

**Results:**

Mutant cyclin F^S621G^ induced cytoplasmic mislocalisation, ER stress (assessed by XBP-1 and CHOP activation), proteasome dysfunction (indicated by GFPu accumulation), and apoptosis (assessed by nuclear condensation and caspase-3 activation), all of which were significantly attenuated by PDI co-expression. Zebrafish larvae expressing cyclin F^S621G^ swam significantly shorter distances than uninjected or wildtype controls, and co-expression of PDI restored motor function to near-wildtype levels.

**Conclusion:**

PDI protects against ALS/FTD-associated mutant cyclin F in both cellular and *in vivo* zebrafish models, identifying PDI as a redox-based therapeutic target for *CCNF*-linked ALS/FTD.

## Introduction

Amyotrophic lateral sclerosis (ALS) is an adult-onset neurodegenerative disorder affecting both upper motor neurons of the motor cortex and lower motor neurons of the brainstem and spinal cord [1,2]. Approximately 10% of all ALS cases are inherited (familial; fALS), caused by mutations in specific genes associated with ALS, while the remaining 90% of cases are sporadic (sALS), where known genetic causes account for 11% of cases [3]. Mutations in the *CCNF* gene, encoding cyclin F, are present in both fALS and sALS [4,5]. Cyclin F forms part of an E3 ubiquitin‒protein ligase complex, which mediates the ubiquitination and proteasomal degradation of target proteins and thus is a fundamental component of the ubiquitin proteasome system (UPS) [6,7]. E3 ubiquitin ligases are also involved in maintaining endoplasmic reticulum (ER) homoeostasis by directing target proteins for degradation via ER-associated degradation (ERAD) [8,9].

The mechanisms underlying ALS/FTD-associated cyclin F cases remain unclear. However, the ALS/FTD-associated mutant cyclin F^S621G^ induces UPS dysfunction, leading to the accumulation of ubiquitinated proteins, including TAR DNA-binding protein 43 (TDP-43) [10], which is found in a pathological form in almost all (97%) ALS and ~50% FTD cases [11,12]. Similar to TDP-43 and related protein Fused in Sarcoma (FUS), mutant cyclin F^S621G^ also mislocalises to the cytoplasm in ALS, whereas wildtype cyclin F is predominately localised in the nucleus [13,14].

Zebrafish expressing mutant cyclin F^S621G^ are a useful tool to examine ALS *in vivo* because they display a motor phenotype in response to a light stimulus (photomotor response) [15]. Mutant cyclin F^S621G^ inhibits ERAD and ER-Golgi protein transport, and induces fragmentation of the Golgi apparatus [16,17], triggering ER stress and apoptosis in neuronal cell lines [16]. ER stress arises due to disturbances in ER homoeostasis, including ERAD and ER-Golgi trafficking defects, leading to excess accumulation of misfolded proteins in the ER lumen [18]. This triggers the unfolded protein response (UPR), an adaptive mechanism designed to restore ER homoeostasis [19]. However, under conditions of chronic or irreversible ER stress, the UPR becomes pro-apoptotic [20–22].

PDI is a chaperone that is primarily localised within the ER and is normally upregulated during ER stress [23]. It is the prototype of a large family of proteins that also possess disulphide interchange activity, whereby PDI can either oxidise, reduce, or isomerise protein disulphide bonds. These functions allow PDI to promote the correct folding of misfolded proteins into their native conformations. Hence, it is a fundamental constituent of the ER proteostasis machinery [24]. However, PDI is also present in other cellular locations, including the cytoplasm and nucleus [25,26], consistent with its known activity in these compartments. Importantly, PDI is protective against the cellular ALS phenotypes induced by mutant SOD1, TDP-43, and FUS, and against motor dysfunction and axonopathy in zebrafish mutant SOD1 models [27,28].

Furthermore, closely related family member PDIA3 (ERp57) prevents ALS-associated mutant TDP-43 defects in neuronal cells [29,30] and motor impairment and early denervation in SOD1^G93A^ mice [31], implying that PDI proteins have broad protective activity in ALS. Consistent with this notion, both PDI and ERp57 mutations are implicated as ALS risk factors [32,33]. However, it remains unknown whether PDI is protective against ALS phenotypes associated with mutant cyclin F. Hence, we tested this hypothesis in this study. We show that PDI prevents multiple phenotypes in mutant cyclin F-associated ALS. In neuronal cells, PDI prevented mis-localisation of cyclin F^S621G^ from the nucleus to the cytoplasm, UPS dysfunction, ER stress, and apoptosis, and in zebrafish expressing cyclin F^S621G^, it prevented motor impairment. This study therefore shows that harnessing the activity of PDI is a novel therapeutic strategy in ALS/FTD-linked to *CCNF* mutations.

## Materials and methods

### Expression constructs

A pcDNA3.1 vector containing full-length PDI and a V5 tag at the C-terminus was kindly provided by Dr. Neil Bulleid, University of Glasgow, UK [34]. Constructs encoding either GFPu, used to indicate activation of the UPS [35], or wildtype cyclin F and mutant cyclin F^S621G^ (in pmCherry.N1) [4], were as previously described. A previously described XBP-1 Venus reporter was used to signal XBP-1 activation and hence, induction of ER stress [36]. All the plasmids were verified by DNA sequencing before use.

### Cell culture and transfection

Mouse neuroblastoma Neuro-2a cells (ATCC cell line CCL-131) were maintained in high-glucose Dulbecco’s Modified Eagle Medium (DMEM with GlutaMAX supplement; Gibco; #10564011) supplemented with 10% (v/v) heat-inactivated foetal calf serum (FCS) and 1% (v/v) penicillin-streptomycin (Gibco; #15070063). The cells were incubated in a humidified 37 °C incubator with 5% CO_2_ and passaged upon 75%–80% confluence. A total of 5 × 10^4^ cells were seeded in each well of a 24-well plate containing 13 mm coverslips (Menzel) to perform immunocytochemistry. A total of 1 × 10^5^ cells were seeded in each well of a 6-well plate to prepare cell lysates. Transfections were performed using Lipofectamine® 2000 with PLUS reagent (Invitrogen, #11668019) according to the manufacturer’s protocol, using 2 µL of Lipofectamine® 2000 per 1 µg of plasmid DNA. Briefly, cells were transfected with 0.5  µg of either wildtype or mutant cyclin F, and 0.5  µg of PDI or empty vector constructs, and incubated for 72 h in a 37 °C incubator with 5% CO_2_. To examine the activation of the UPS or XBP-1, 0.5  µg of GFPu or XBP-1-Venus reporter was also co-transfected, respectively, where indicated.

### Immunocytochemistry

Transfected Neuro-2a cells grown on coverslips were fixed with 200 µL of 4% (w/v) paraformaldehyde in phosphate-buffered saline (PBS; Gibco, #10010023), prepared by diluting 16% (w/v) paraformaldehyde aqueous solution (ProSciTech, #C004) 1:4 in PBS, for 30 min at room temperature in the dark. Following fixation, the cells were washed three times with PBS before permeabilisation with 200 µL of 0.1% (v/v) Triton X-100 (Sigma–Aldrich, #X100) in PBS for 10 min at room temperature. Cells were then blocked with 200 µL of 3% (w/v) bovine serum albumin (BSA; Sigma–Aldrich, #A7030) in PBS for 1 h at room temperature. Following blocking, the cells were washed twice with PBS for 10 min per wash. Cells were then incubated with 100 µL of the appropriate primary antibody diluted in PBS overnight at 4 °C. The primary antibodies used were mouse anti-GADD153/CHOP (1:500; Santa Cruz Biotechnology, #sc-7351), rabbit anti-calreticulin (1:200; Abcam, #ab2907), rabbit anti-cleaved caspase-3 (Asp175; 1:500; Cell Signaling Technology, #9661), mouse anti-XBP1 (1:500; Santa Cruz Biotechnology, #sc-8015), or rabbit anti-mCherry (EPR20579; 1:500; Abcam, #ab213511) to detect cyclin F-mCherry, and mouse anti-V5 (1:500; Invitrogen, #R960-25) to detect PDI-V5. Cells were washed three times with PBS for 10 min per wash before incubation with 100 µL of the appropriate Alexa Fluor-conjugated secondary antibody diluted in PBS for 1 h at room temperature in the dark. The secondary antibodies used were goat anti-mouse IgG Alexa Fluor 488 conjugate (1:250; Invitrogen, #A-11001) and goat anti-rabbit IgG Alexa Fluor 488 conjugate (1:250; Invitrogen, #A-11008). Cells were further washed three times with PBS for 10 min per wash, and the nuclei were stained with Hoechst 33342 (Sigma–Aldrich, #14533), diluted 1:3000 in PBS, for 7 min at room temperature in the dark. Coverslips were mounted onto microscope slides using fluorescent mounting medium (15  µl; Dako, # S3023) and allowed to air-dry in the dark overnight.

### Nuclear morphology analysis to identify apoptotic cells

Neuro-2a cells fixed with 4% (w/v) PFA for 30 min in the dark at room temperature were washed and treated with Hoechst 33342 (0.5  µg/mL; Sigma, #14533) for 7 min in the dark at room temperature. After two washes with PBS, the cells were mounted with Dako fluorescent mounting medium. Images were acquired using a Zeiss AxioImager epifluorescence microscope and accompanying Zen software. At least 100 cells co-expressing both transfected plasmids per treatment were analysed, from three independent experiments. Nuclei were identified as being apoptotic when they were either condensed (≤ 5 μm in diameter) or fragmented (numerous condensed Hoechst-positive structures in a single cell), as previously described [25,27,37]. Using ImageJ software (v. 1.53) (NIH), the percentage of cells with apoptotic nuclei was quantified from at least 100 cells expressing the desired plasmid. Cells which were undergoing cell division were omitted from the study.

### Microscopy

The slides were examined using 40x magnification on a Zeiss AxioImager epifluorescence microscope. DAPI (nuclei, blue fluorescence), Fluorescein isothiocyanate (FITC, green fluorescence), and TRITC (red fluorescence) filters with Zen software were used for cell counting and analysis. Photomultiplier sensitivities were set to minimise ‘bleed through’ effects from one channel to another. At least 100 cells co-expressing both transfected plasmids were counted and analysed per treatment. Confocal imaging was performed using a Zeiss LSM 880 confocal laser scanning microscope for the attainment of high-resolution images.

For cyclin F localisation analysis, only cells co-expressing cyclin F-mCherry and the relevant co-transfected protein were included. Cyclin F localisation was assessed using predefined criteria and quantified with the assistance of ImageJ/Fiji software. Cyclin F was classified as nuclear when the mCherry signal overlapped with the Hoechst-positive nucleus, and as cytoplasmic when mCherry fluorescence was detected in the cytoplasm, either with or without a nuclear signal. At least 100 co-transfected cells per condition were analysed from three independent experiments, and quantification was performed in a blinded manner to the experimental group.

### Subcellular fractionation

Neuro-2a cells were transfected with mCherry-tagged wildtype cyclin F or mutant cyclin F^S621G^ in the presence or absence of V5-tagged PDI. Twenty-four hours post-transfection, the cells were lysed in fractionation buffer containing 20 mM HEPES, pH 7.4, 10 mM KCl, 1.5 mM MgCl₂, and 1 mM EDTA. Following centrifugation at 720 × g for 5 min, the supernatant containing the cytoplasmic-enriched fraction was transferred into fresh tubes. The pellet containing the nuclear fraction was washed with 500 µL of fractionation buffer and centrifuged twice at 720 × g for 10 min. The nuclear pellet was then resuspended in 200 µL of RIPA buffer containing 0.1% SDS, followed by brief sonication for 10 s on ice to shear genomic DNA and homogenise the lysate. The cytoplasmic-enriched supernatant was centrifuged at 10,000 × g for 5 min, and the resulting supernatant was collected and stored at −20 °C until required.

### Western blotting

The total protein concentration in each sample was quantified using a Pierce BCA Protein Assay Kit (Thermo Fisher Scientific, #23225), according to the manufacturer’s instructions. Protein samples were separated by SDS–PAGE using either 7.5% or 4%–15% Mini-PROTEAN TGX gels (Bio-Rad) and transferred onto nitrocellulose membranes according to the manufacturer’s instructions. The membranes were blocked and incubated with primary antibodies against mCherry to detect cyclin F-mCherry, V5 to detect PDI-V5, lamin B as a nuclear marker, and GAPDH as a cytoplasmic marker. The primary antibodies used were rabbit anti-mCherry (1:2000; Abcam, #ab213511), mouse anti-V5 (1:2000; Invitrogen, #R960-25), rabbit anti-lamin B1 (1:3000; Abcam, #ab16048), and mouse anti-GAPDH (1:4000; Proteintech, #60004-Ig). Following primary antibody incubation, the membranes were washed three times with Tris-buffered saline containing 0.1% Tween-20 (TBST) for 10 min per wash. The membranes were then incubated with the appropriate horseradish peroxidase-conjugated secondary antibodies (1:2000; Millipore #71-045-3MI) for 1 h at room temperature. After secondary antibody incubation, the membranes were further washed three times with TBST for 10 min per wash. Immunoreactivity was detected using the Clarity ECL Western Blotting Substrate kit (Bio-Rad, #1705061), and images were acquired using a Bio-Rad ChemiDoc MP imaging system with Image Lab software (Bio-Rad). Band intensities were quantified using ImageJ software (v.1.47; National Institutes of Health). The Cyclin F-mCherry signal in the cytoplasmic fraction was normalised to GAPDH for the cytoplasmic fraction and normalised to lamin B1 for the nuclear fraction.

### Zebrafish maintenance and mRNA microinjection

All animal husbandry and experimental procedures were performed in compliance with the Animal Ethics Committee, Macquarie University, NSW, Australia (ARA 2015/034 and ARA 2017/019) and the Internal Biosafety Committee, Macquarie University NLRD5974 (52019597412350). Transgenic zebrafish (*Danio rerio*), expressing blue fluorescent protein (BFP) in their motor neurons, on a TAB-WT background, Tg(-7933mnx1: TagBFP) mq, were bred and maintained under standard zebrafish housing conditions. Adult zebrafish were mated, and the resulting embryos were collected for microinjection experiments. Following spawning, fertilised embryos were pooled and randomly allocated to the experimental groups prior to microinjection. Human wildtype or mutant cyclin F^S621G^ mRNA (100 ng/μl) was injected, along with either mKate2 mRNA or mRNA encoding PDI fused to mKate2 (200 ng/μl), depending on the experimental group, via 0.905 nl droplets into 1–4 cell stage zebrafish embryos using a Picospritzer II (Parker Instrumentation). At 24 h post-fertilisation (hpf), the embryos were screened for successful injection (expression of mKate2 red fluorescent protein) using an M165FC fluorescent stereomicroscope (Leica). Embryos exhibiting developmental abnormalities were excluded at this stage, and only morphologically normal larvae expressing mKate2 were retained for subsequent analysis. These inclusion and exclusion criteria were established *a priori*. Positive embryos were manually dechorionated (shells were removed) with forceps and raised in equal numbers in E3 medium (5  mM NaCl, 0.17  mM KCl, 0.33  mM CaCl2 and 0.33  mM MgSO4) in darkness at 28 °C.

The group allocation was blinded throughout the *in vivo* experiments. An independent researcher coded the experimental groups, and the investigators who performed the behavioural tracking and quantification were blinded to group identity until analysis was complete.

### Tracking the movement of zebrafish embryos

At 48 hpf, morphologically normal larvae were distributed into a 96-well plate, where each embryo was placed into a separate well containing 250 μl of E3 medium. The micro-well plate was then incubated at 28 °C for 15 min prior to examining motor function. Tracking of zebrafish embryo movement was performed using a Zebrabox (Viewpoint) automated recording device, as described previously [27]. The 96-well plates containing zebrafish larvae were placed inside the Zebrabox device and left for 30 min to allow them to acclimatise to the dark. The motor response to a flash of light (1-second flash of 300 watts) was then examined three times at 1-minute intervals. The distance travelled by each animal during the 3 min, starting from the first flash of light, was recorded.

The experimental unit was a single larva. In total, 242 larvae were analysed across the four groups (uninjected, *n* = 55; wildtype cyclin F, *n* = 55; mutant cyclin F^S621G^, *n* = 66; and mutant cyclin F^S621G^ co-injected with PDI, *n* = 66), pooled from three independent biological replicates. The differing group sizes reflect the exclusion of embryos that did not pass the screening criteria described above. No *a priori* sample size calculation was performed; group sizes were based on those used in previous zebrafish photomotor studies of ALS/FTD that reliably detected motor phenotypes [15].

Following behavioural analysis, zebrafish larvae (48 hpf, <5 dpf) were euthanised by immersion in dilute sodium hypochlorite (6.15% stock diluted 1:5 in system water, final concentration >1%) for a minimum of 5 min prior to disposal, in accordance with the AVMA Guidelines for the Euthanasia of Animals (2020 Edition).

### Statistical analysis

Each experiment was performed blinded a minimum of three independent times, unless otherwise stated. Data normality was assessed using the Shapiro‒Wilk test. For cellular and biochemical datasets that passed normality testing, comparisons between multiple groups were performed using one-way analysis of variance (ANOVA) followed by Tukey’s multiple comparisons post hoc test. Zebrafish swimming behaviour was analysed using a mixed-effects model to account for variability between independent experimental runs, followed by Tukey’s multiple comparisons post hoc test. All analyses were performed using GraphPad Prism 10.2 (San Diego, CA, U.S.A.). Data are presented as the mean ± standard error of the mean (SEM). *P*-values < 0.05 were considered statistically significant, where *p < 0.05, **p < 0.01, ***p < 0.001 and ****p < 0.0001.

## Results

### Overexpression of PDI inhibits mutant cyclin F localisation to the cytoplasm

Western blotting was performed to validate expression of the transfected constructs used in the cellular experiments. Cell lysates were prepared from Neuro-2a cells expressing the mCherry vector alone, wildtype cyclin F, wildtype cyclin F with PDI-V5, mutant cyclin F^S621G^, or mutant cyclin F^S621G^ with PDI-V5. Western blotting confirmed the expression of V5-tagged PDI in the PDI-transfected groups, but not in the corresponding control groups. Cyclin F-mCherry was detected in cells expressing wildtype or mutant cyclin F constructs, while mCherry alone was detected in the mCherry vector control (Supplementary Figure 1A). *β*-actin was used as a loading control. These data confirm successful expression of the cyclin F and PDI-V5 constructs used in the cellular assays and they show that PDI-V5 overexpression did not markedly alter cyclin F expression levels (Supplementary Figure 1A).

Neuro-2a cells were selected because of their relatively high transfection efficiency compared to other neuronal cell lines. This is important because co-transfection with PDI and reporter constructs with cyclin F was performed; hence, a high transfection efficiency was essential. The co-transfection efficiency of PDI and cyclin F constructs was previously validated in our earlier studies examining the protective effects of PDI in ALS models, where high transfection efficiency and consistent expression of PDI constructs were confirmed [25,27]. Similar cyclin F expression approaches have also been previously established in mutant cyclin F cellular models [16]. Therefore, subsequent experiments were performed using these previously validated conditions.

While cyclin F is normally localised in the nucleus, it has been previously established that mutant cyclin F^S621G^ mislocalises to the cytoplasm in ALS, similar to TDP-43 and FUS [13]. Therefore, it was investigated whether PDI could prevent mutant cyclin F^S621G^ mislocalisation to the cytoplasm in Neuro-2a cells. Cells transfected as described above were subjected to immunocytochemistry using an anti-V5 antibody to detect PDI expression and stained with Hoechst to clearly visualise the nuclei. Cells were examined for cyclin F localisation using confocal microscopy. Cyclin F was considered nuclear when it was completely co-localised with the Hoechst-stained nucleus, but it was categorised as cytoplasmic when fluorescence was detected either in both the nucleus and the cytoplasm, or within the cytoplasm only, of cyclin F-expressing cells (Figure 1A).

**Figure 1.**
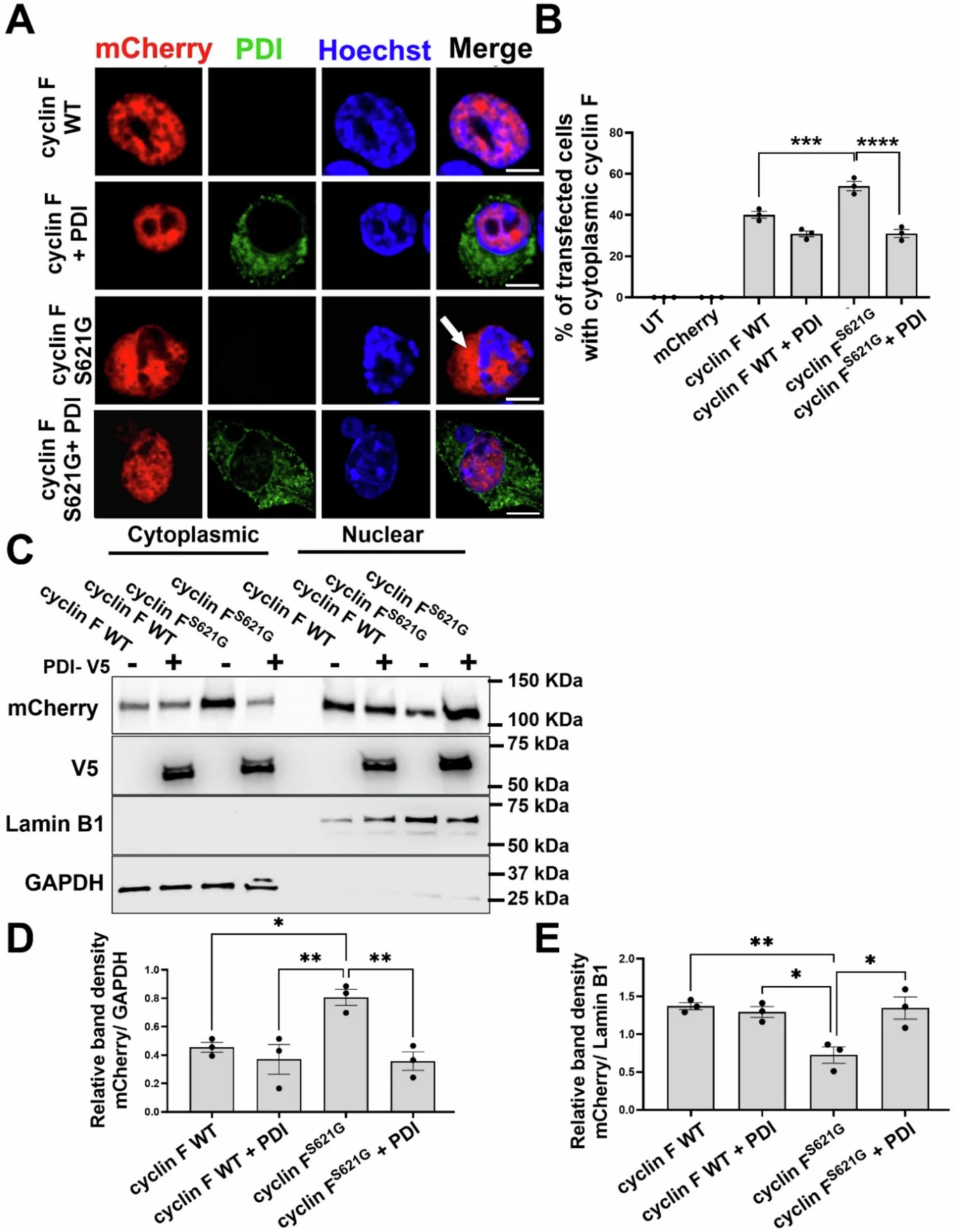
Overexpression of PDI inhibits ALS-mutant cyclin F^S621G^ localisation to the cytoplasm. (A) Confocal images of Neuro-2a cells expressing either wildtype or mutant cyclin F^S621G^ (first column), with or without co-expression of PDI (second column). Hoechst-stained nuclei are illustrated in column three, and a merged image of the cell is shown in column four. Cytoplasmic localisation of ALS-mutant cyclin F^S621G^ is indicated with a white arrow. Scale bar = 30 µm. (B) Quantification of Neuro-2a cells with cytoplasmic localisation of cyclin F from the images in A). There was a significant increase (***p < 0.001) in the proportion of cells with cytoplasmic localisation of mutant cyclin F^S621G^, compared to wildtype cyclin F. However, significantly fewer cells with cytoplasmic localisation of cyclin F were detected when mutant F^S621G^, but not wildtype, was co-expressed with PDI (****p < 0.0001). (C) Representative immunoblots of cytoplasmic and nuclear fractions from Neuro-2a cells expressing mCherry-tagged wildtype cyclin F or mutant cyclin F^S621G^, in the presence or absence of V5-tagged PDI. Cyclin F-mCherry was detected using an anti-mCherry antibody, and PDI overexpression was confirmed using an anti-V5 antibody. GAPDH and lamin B1 were used as cytoplasmic and nuclear fraction markers, respectively. (D) Quantification of cyclin F-mCherry expression in the cytoplasmic fraction of the blots in (C), normalised to GAPDH. Mutant cyclin F^S621G^ showed increased cytoplasmic abundance compared with wildtype cyclin F (*p < 0.05), while co-expression of PDI significantly reduced the proportion of mutant cyclin F^S621G^ in the cytoplasm (**p < 0.01). (E) Quantification of cyclin F-mCherry expression in the nuclear fraction, normalised to lamin B1. Mutant cyclin F^S621G^ showed less nuclear abundance compared with wildtype cyclin F (**p < 0.01), while co-expression of PDI significantly increased mutant cyclin F^S621G^ in the nucleus (*p < 0.05). *N *= 3 independent experiments. Data are presented as mean ± SEM, *p < 0.05, **p < 0.01, ***p < 0.001, ****p < 0.0001.

A significantly higher proportion of cells expressing mutant cyclin F^S621G^ (***p < 0.001) displayed cytoplasmic cyclin F (54.3% ± 3.4), compared to cells expressing wildtype cyclin F. However, co-overexpression of PDI resulted in significantly (****p < 0.0001) fewer cells with cytoplasmic cyclin F localisation (30.7% ± 2.9), compared to cells co-expressing mutant cyclin F^S621G^ with empty vector pcDNA3.1 (Figure 1B). Wildtype cyclin F was localised in the cytoplasm in 41.0% ± 2.5 of cells expressing cyclin F, and this proportion was decreased following co-expression of PDI (to 31.0% ± 2.0 cells), although this difference did not reach statistical significance. Therefore, these data reveal that overexpression of PDI is protective against the cytoplasmic mislocalisation of ALS-associated mutant cyclin F^S621G^.

To further validate the microscopy-based localisation findings, nuclear and cytoplasmic biochemical fractionation was performed in Neuro-2a cells expressing mCherry-tagged wildtype cyclin F or mutant cyclin F^S621G^ in the presence or absence of V5-tagged PDI. Western blotting confirmed the expression of mCherry-tagged cyclin F in the relevant transfected groups and robust expression of PDI-V5 in PDI-transfected cells, but not in empty vector controls (Figure 1C). GAPDH was enriched in the cytoplasmic fraction, and lamin B1 was enriched in the nuclear fraction, confirming successful separation of the cytoplasmic and nuclear fractions (Figure 1C).

Quantification of mCherry-tagged cyclin F in the cytoplasmic fraction, normalised to GAPDH, showed that mutant cyclin F^S621G^ was significantly increased in the cytoplasm compared with wildtype cyclin F (*p < 0.05; Figure 1D). Co-expression of PDI significantly reduced the cytoplasmic abundance of mutant cyclin F^S621G^ compared with that of mutant cyclin F^S621G^ alone, which was now similar to wildtype cyclin F (**p < 0.01; Figure 1D). In contrast, PDI did not markedly alter cytoplasmic wildtype cyclin F levels.

Similarly, quantification of mCherry-tagged cyclin F in the nuclear fraction, normalised to lamin B1, showed that the level of mutant cyclin F^S621G^ was significantly reduced in the nucleus compared with wildtype cyclin F (**p < 0.01; Figure 1E). Co-expression of PDI significantly increased nuclear mutant cyclin F^S621G^ compared with mutant cyclin F^S621G^ alone (*p < 0.05; Figure 1E), restoring nuclear cyclin F abundance similar to wildtype levels. Together, these biochemical fractionation data support the confocal microscopy findings and indicate that PDI attenuates cytoplasmic mislocalisation of ALS/FTD-associated mutant cyclin F^S621G^.

### Overexpression of PDI is protective against ER stress induced by mutant cyclin F

It was previously demonstrated that ALS-associated cyclin F induces ER stress in neuronal cell lines using two markers: XBP-1 and CHOP [16]. The activation of XBP-1 was used as a marker of UPR induction in its early, pro-survival stages, reflecting activation of the inositol-requiring enzyme 1 (IRE-1) pathway [38,39]. In contrast, CHOP is a marker induced via the PERK arm of the UPR, and it is activated at a later phase than XBP-1 [20], reflecting the cellular transition from the pro-survival to pro-apoptotic phases of the UPR. Therefore, nuclear immunoreactivity to CHOP, signifying CHOP activation, is a useful marker to indicate that ER stress-induced apoptotic signalling has been induced within cells [16].

A previously described reporter construct for XBP-1 was used to detect its activation [36,40]. XBP-1-Venus contains the GFP variant, Venus, downstream of a partial sequence of XBP-1. Under normal, non-stressed conditions, the mRNA generated from this construct will not be spliced, and translation will be terminated at a stop codon between the XBP-1 and Venus genes. Hence, no fluorescence is generated. Conversely, during ER stress, this mRNA is spliced, leading to a frameshift, which results in termination of translation at the stop codon after Venus. Thus, a fusion protein of XBP-1 and Venus will be produced in cells undergoing ER stress, detected by Venus fluorescence in the nucleus due to XBP-1 activation. Hence, nuclear immunoreactivity to XBP-1 is a useful marker of its activation, and thus, ER stress [36] (Figure 2A).

**Figure 2.**
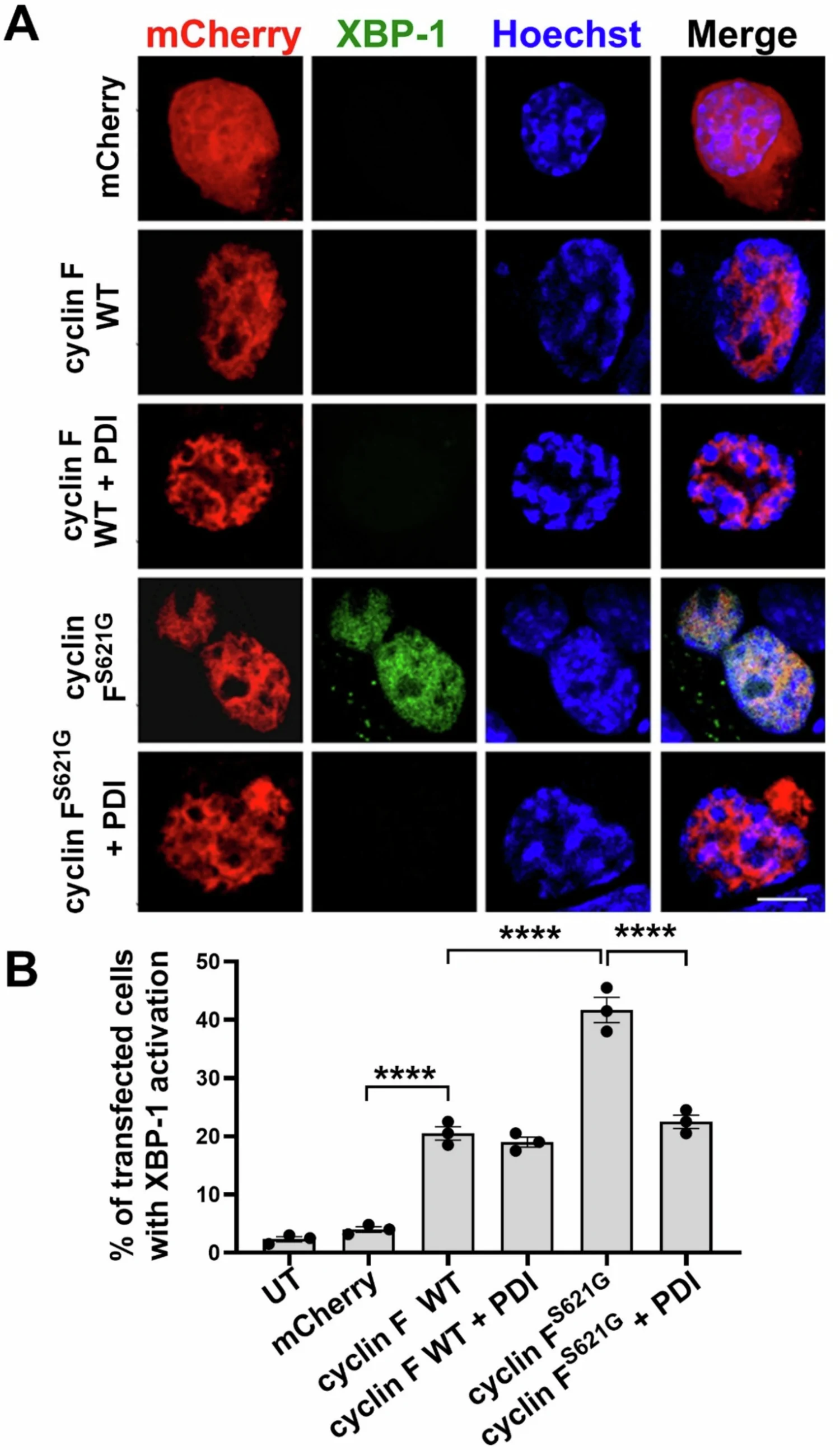
Overexpression of PDI is protective against XBP-1 activation induced by mutant cyclin F. (A) Confocal images of Neuro-2a cells expressing mCherry alone and either wildtype or mutant cyclin F^S621G^ (first column), with or without co-expression of PDI-V5 (second column). Scale bar = 30 µm. (B) Quantification of cells from the images in A) with XBP-1 nuclear immunoreactivity. The proportion of cells with XBP-1 activation was significantly higher in cells expressing cyclin F^S621G^ compared to wild-type cyclin F (****p < 0.0001). When PDI was co-expressed with cyclin F^S621G^, significantly fewer cells (****p < 0.0001) displayed XBP-1 activation, implying that PDI is protective against ER stress induced by mutant cyclin F, but not by wildtype cyclin F. *N* = 3, mean ± SEM, ****p < 0.0001.

Neuro-2a cells were co-transfected with constructs encoding the XBP-1-Venus reporter, either the mCherry-tagged wildtype or mutant cyclin F^S621G^-mCherry, and either the pcDNA3.1 empty vector (as a control) or PDI-V5 pcDNA3.1. mCherry-only and XBP-1 Venus-only controls were included to ensure that these proteins alone did not induce ER stress. After 72 h of transfection, Hoechst staining and fluorescent microscopy were performed. In untransfected cells and cells transfected with mCherry alone, very little XBP-1 activation (<5% cells) was detected. Mutant cyclin F^S621G^ cells co-expressing empty vector displayed significantly more (**** *p* < 0.0001) XBP-1 activation (41.3% ± 2.7) compared to cells co-expressing wildtype cyclin F with the empty vector (20.7% ± 3.3). Moreover, co-expression of mutant cyclin F^S621G^ with PDI significantly reduced (****p < 0.0001) the proportion of cells with XBP-1 activation to 22.7% ± 1.8, similar to the levels seen in cells expressing wildtype cyclin F (Figure 2B). Also, XBP-1 activation was detected in more cells expressing wildtype cyclin F (20.7% ± 3.3) compared to mCherry alone (****p < 0.0001). However, PDI did not inhibit XBP-1 activation in wildtype cyclin F cells (19.3% ± 1.2). Hence, these results indicate that the overexpression of PDI is protective against XBP-1 nuclear immunoreactivity, and hence ER stress, induced by the ALS-associated mutant cyclin F^S621G^.

CHOP is a transcription factor that translocates to the nucleus during ER stress [41–43]. Hence, nuclear immunoreactivity to CHOP can be used to indicate the activation of the pro-apoptotic phase of the UPR [42–44] (Figure 3A). Next, cells co-expressing wildtype or mutant cyclin F with PDI-V5 or an empty vector for 72 h were subjected to immunocytochemistry using an anti-CHOP antibody with Hoechst staining to visualise the nuclei. Quantification revealed that untransfected cells and cells expressing mCherry alone displayed little nuclear immunoreactivity to CHOP (3.0% ± 0.6 and 2.3% ± 0.3 of cells, respectively), as expected. There was a significant increase (****p < 0.001) in the proportion of cells with nuclear immunoreactivity to CHOP in populations expressing mutant cyclin F^S621G^ (35.7% ± 3.0), compared to wildtype cyclin F cells. In contrast, co-expression of PDI with cyclin F^S621G^ resulted in significantly fewer (***p < 0.01) cells with CHOP activation (23.7% ± 1.2), compared to those co-expressing cyclin F^S621G^ with the empty vector. There was also a significant (****p < 0.0001) increase in the proportion of cells displaying CHOP activation in cells expressing wildtype cyclin F (14.7% ± 1.5), as for XBP-1. Similarly, co-expression of PDI with wildtype cyclin F did not alter the proportion of cells with CHOP activation (12.3% ± 1.2) (Figure 3B). Hence, these findings imply that PDI is protective against ER stress-induced apoptotic signalling induced by ALS/FTD-associated mutant, but not wildtype, cyclin F in Neuro-2a cells.

**Figure 3.**
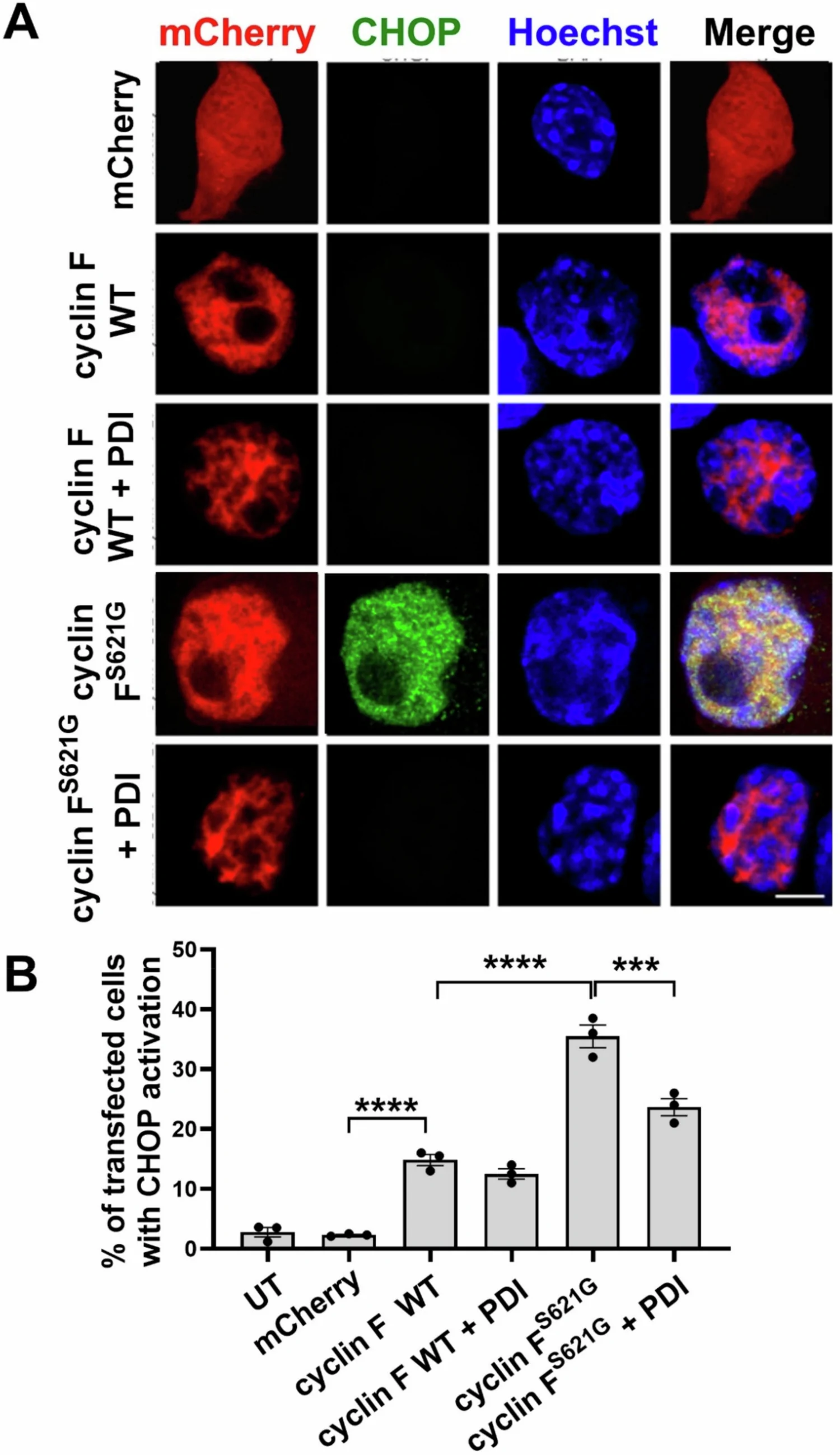
Overexpression of PDI is protective against CHOP activation induced by mutant cyclin F. (A) Neuro-2a cells were transfected with mCherry alone (first panel), wildtype cyclin F co-expressed with either empty vector (second panel; cyclin F WT) or PDI-V5 (third panel), or mutant cyclin F^S621G^ co-expressed with either empty vector (fourth panel) or PDI-V5 (fifth panel). Scale bar = 30 µm. (B) Quantification of cells from the images in A) displaying CHOP activation. Very few untransfected (UT) cells and cells expressing mCherry alone displayed nuclear CHOP immunoreactivity. There was a significant increase (****p < 0.0001) in the proportion of wildtype cyclin F cells compared to mCherry cells with CHOP activation. Significantly more (****p < 0.0001) cells expressing cyclin F^S621G^ displayed nuclear immunoreactivity to CHOP, compared to cells expressing wildtype cyclin F. However, significantly fewer (***p < 0.001) cyclin F^S621G^ cells displayed CHOP activation when PDI was co-expressed, compared to those expressing empty vector. *N* = 3, Mean ± SEM, ***p < 0.001, ****p < 0.0001.

### Overexpression of PDI inhibits dysfunction of the UPS induced by mutant cyclin F

As ALS/FTD-associated mutant cyclin F impairs ubiquitin-mediated proteasomal degradation in neuronal cells [4], it was next examined whether PDI could prevent UPS dysfunction induced by mutant cyclin F using a previously described reporter, GFPu [45]. This construct consists of a 16 amino acid degron (CL1, a specific substrate for the UPS), fused to the carboxyl terminus of GFP [46]. Normally, GFP is degraded by the UPS via this degron. Therefore, the accumulation of GFPu (visualised by microscopy) is indicative of UPS impairment [46]. Wildtype and mutant cyclin F-mCherry were co-expressed with GFPu, and either empty vector pcDNA3.1 or V5-tagged PDI for 72 h. Cells were analysed using confocal microscopy, and the percentage of cyclin F-expressing cells with clearly visible GFPu expression (indicated by green fluorescence) was quantified (Figure 4A).

**Figure 4.**
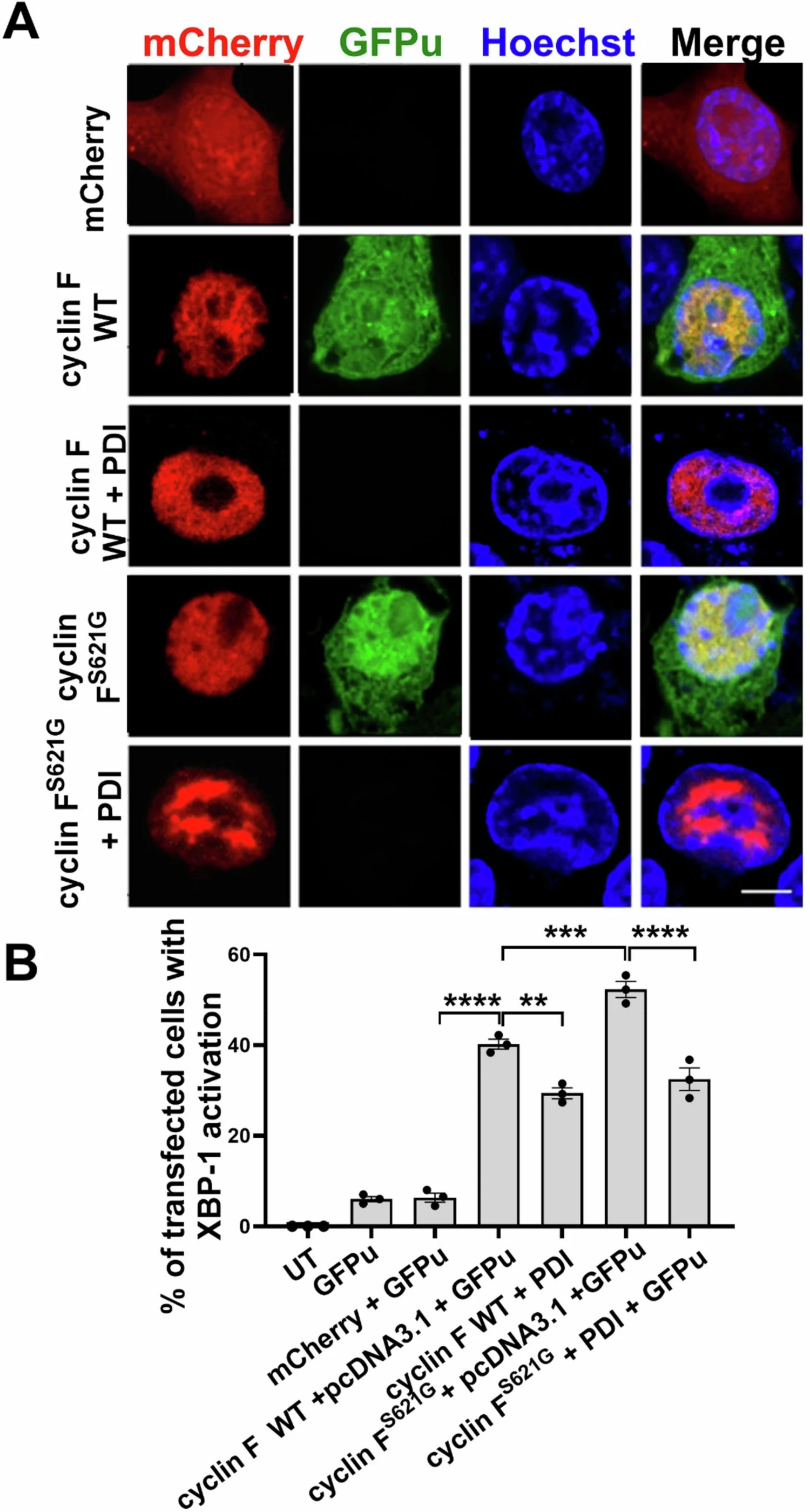
Overexpression of PDI inhibits UPS dysfunction induced by mutant and wildtype cyclin F. (A) Neuro-2a cells expressing mCherry alone (first panel), wildtype cyclin F co-expressed with either an empty vector (second panel; cyclin F WT) or PDI-V5 (third panel), or mutant cyclin F^S621G^ co-expressed with either an empty vector (fourth panel) or PDI-V5 (fifth panel). Scale bar = 30 µm. (B) Quantification of cells from the images in A). Very few untransfected (UT) and mCherry-expressing cells displayed activation of GFPu. There was a significant increase (***p < 0.001) in the proportion of cells with GFPu activation in populations expressing mutant cyclin F^S621G^, compared to wild-type cyclin F. Moreover, this proportion was significantly reduced (****p < 0.0001) when PDI was co-expressed with mutant cyclin F^S621G^. Significantly more cells (****p < 0.0001) expressing wildtype cyclin F demonstrated UPS dysfunction as detected by GFPu activation, but this proportion was significantly decreased (**p < 0.01) when PDI was co-expressed. *N* = 3, Mean ± SEM, **p < 0.01, ***p < 0.001, ****p < 0.0001.

Very few cells expressing mCherry, pcDNA3.1 vector, or GFPu alone displayed GFPu expression (<10%), demonstrating that the proteasome is functioning normally in these cells. Significantly more cells with GFPu fluorescence were detected in populations expressing mutant cyclin F^S621G^ (52.0% ± 2.6, ***p < 0.001) compared to those expressing wildtype cyclin F. However, following co-expression with PDI, the proportion of cells with GFPu fluorescence was significantly reduced in mutant cyclin F cells (31.7% ± 4.5, ****p < 0.0001) (Figure 4B). An increase in the proportion of cells expressing wildtype cyclin F with GFPu fluorescence (40.3% ± 1.8) was observed compared to mCherry only (****p < 0.0001) indicating that the degradation of GFPu was also impaired in these cells, although to a lower extent than mutant cyclin F^S621G^. However, following co-expression with PDI, there was a significant decrease (**p < 0.01) in the proportion of wildtype cyclin F cells with GFPu activation (29.3% ± 2.2).

### PDI overexpression inhibits mutant cyclin F-induced apoptosis

The activation of CHOP in cells expressing mutant cyclin F^S621G^ compared to control cells implies that the ALS/FTD mutation triggers apoptotic cell death. Hence, next it was examined whether the overexpression of PDI was protective against apoptosis induced by mutant cyclin F. Nuclei condensation is a widely used marker of apoptosis that was examined first in cells expressing mutant cyclin F^S621G^ [25,27,37].

Neuro-2a cells were transfected with wildtype or mutant cyclin F^S621G^ mCherry with either the pcDNA3.1 empty vector or PDI-V5 for 72 h as above. After Hoechst staining, nuclear morphology was examined, where cells were considered apoptotic if the nucleus was condensed (under ~5 µM in diameter) or fragmented (multiple condensed Hoechst-positive structures in one cell), as previously described [44,47] (Figure 5A). Dividing cells were excluded from the study, because they can sometimes appear condensed.

**Figure 5.**
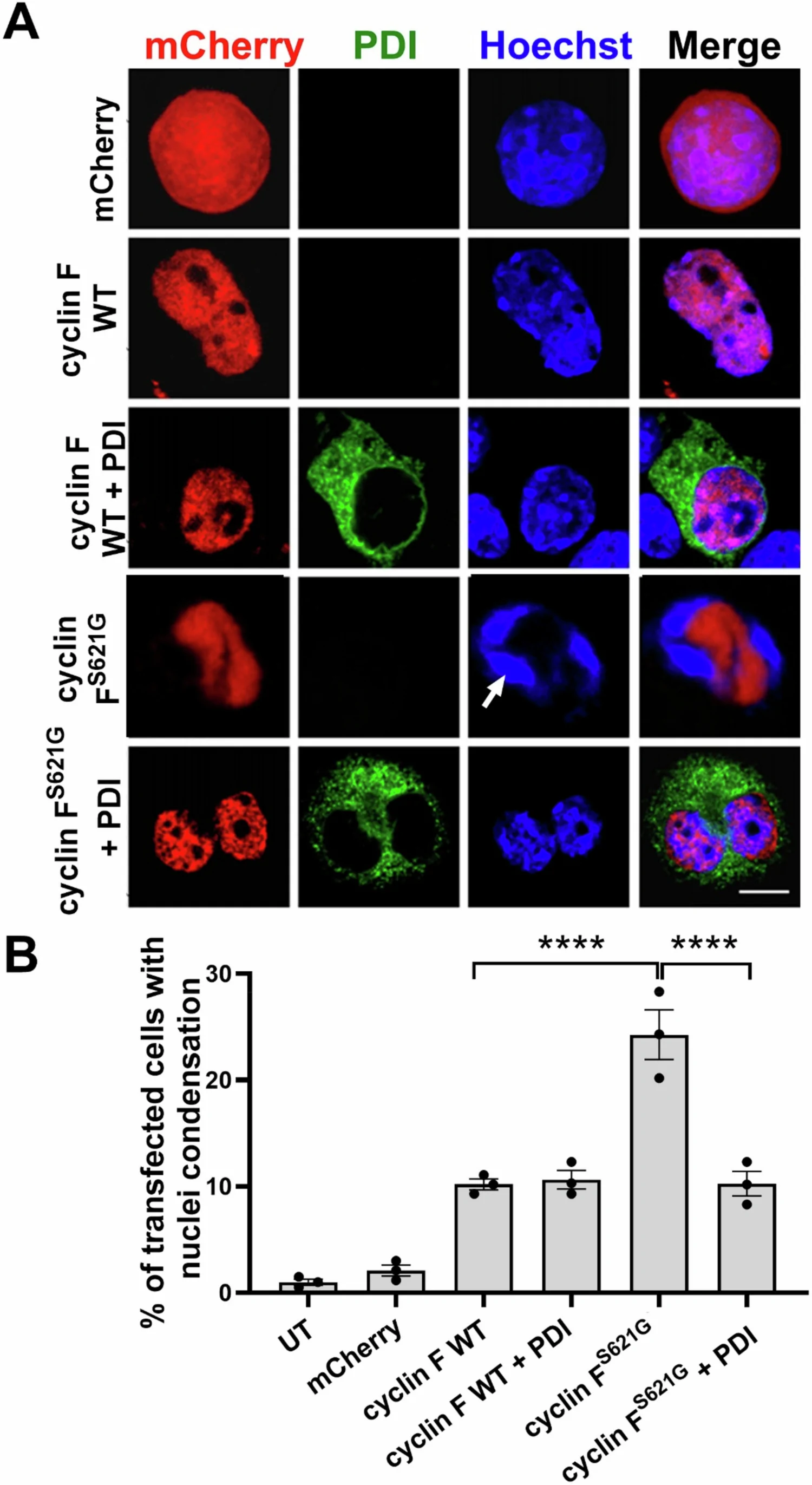
PDI overexpression inhibits mutant cyclin F induced nuclei condensation, indicative of apoptosis. (A) Neuro-2a cells expressing mCherry alone (first panel), wildtype cyclin F co-expressed with either an empty vector (second panel; cyclin F WT) or PDI-V5 (third panel), or mutant cyclin F^S621G^ co-expressed with either an empty vector (fourth panel) or PDI-V5 (fifth panel). Scale bar = 30 µm. (B) Quantification of cells exhibiting apoptotic nuclei in A. More cells with condensed nuclei were present in the mutant cyclin F^S621G^ populations, as indicated with the white arrow, compared to those expressing wildtype cyclin F or mCherry (****p < 0.0001). When PDI was co-expressed with cyclin F^S621G^, however, there was a significant decrease in the proportion of cells with apoptotic nuclei (****p < 0.0001) to similar levels as those present in wildtype cyclin F populations. *N* = 3, mean ± SEM, ****p < 0.0001.

Apoptotic nuclei were rarely present in untransfected cells or cells expressing mCherry alone (1.0% ± 0.6 and 2.0% ± 0.6 cells, respectively). Approximately 10% of cells co-expressing wildtype cyclin F with either the empty vector pcDNA3.1 or PDI displayed condensed nuclei, but this was not significantly different from controls. As previously described, apoptotic nuclei were more common in cells expressing mutant cyclin F^S621G^ (24.0% ± 3.5), compared to those expressing wildtype cyclin F (****p < 0.0001) [25,27,37]. However, significantly fewer (****p < 0.0001) apoptotic nuclei were detected in cells co-expressing PDI with mutant cyclin ^S621G^ (10.0% ± 1.5). Hence, these findings indicate that PDI overexpression is protective against mutant cyclin F-induced apoptosis (Figure 5B).

To confirm these findings, another marker of apoptosis was then examined. Caspase-3 is activated during apoptotic signalling, following the activation of BCL-2-associated X protein (Bax) and the release of cytochrome c from mitochondria, by the cleavage of its inactive form, pro-caspase-3 [48]. Hence, caspase-3 activation was next examined by immunocytochemistry using an antibody specific for cleaved activated caspase-3 (Asp175, Cell Signaling, **#**9661). Neuro-2a cells were co-transfected as described above for 72 h (Figure 6A), and nuclear immunoreactivity to cleaved caspase-3 was examined using confocal microscopy. Less than 10% of untransfected cells or cells expressing mCherry displayed caspase-3 activation, but there was a significant increase in this proportion in cells expressing mutant cyclin F^S621G^ (56.0% ± 3.1) compared to cells expressing wildtype cyclin F (****p < 0.0001). However, when PDI was co-expressed with the mutant cyclin F^S621G^, significantly fewer cells displayed caspase-3 activation (38.7% ± 4.4 of cells, ***p < 0.001). Compared to mCherry alone, significantly more (***p < 0.001) cells expressing wildtype cyclin F displayed activation of caspase-3 (31.3% ± 6.0), but this proportion did not significantly alter when PDI was co-expressed (37.0% ± 1.7). Consistent with the condensed nuclei results, these data reveal that PDI is protective against apoptosis induced by mutant, but not wildtype, cyclin F (Figure 6B).

**Figure 6.**
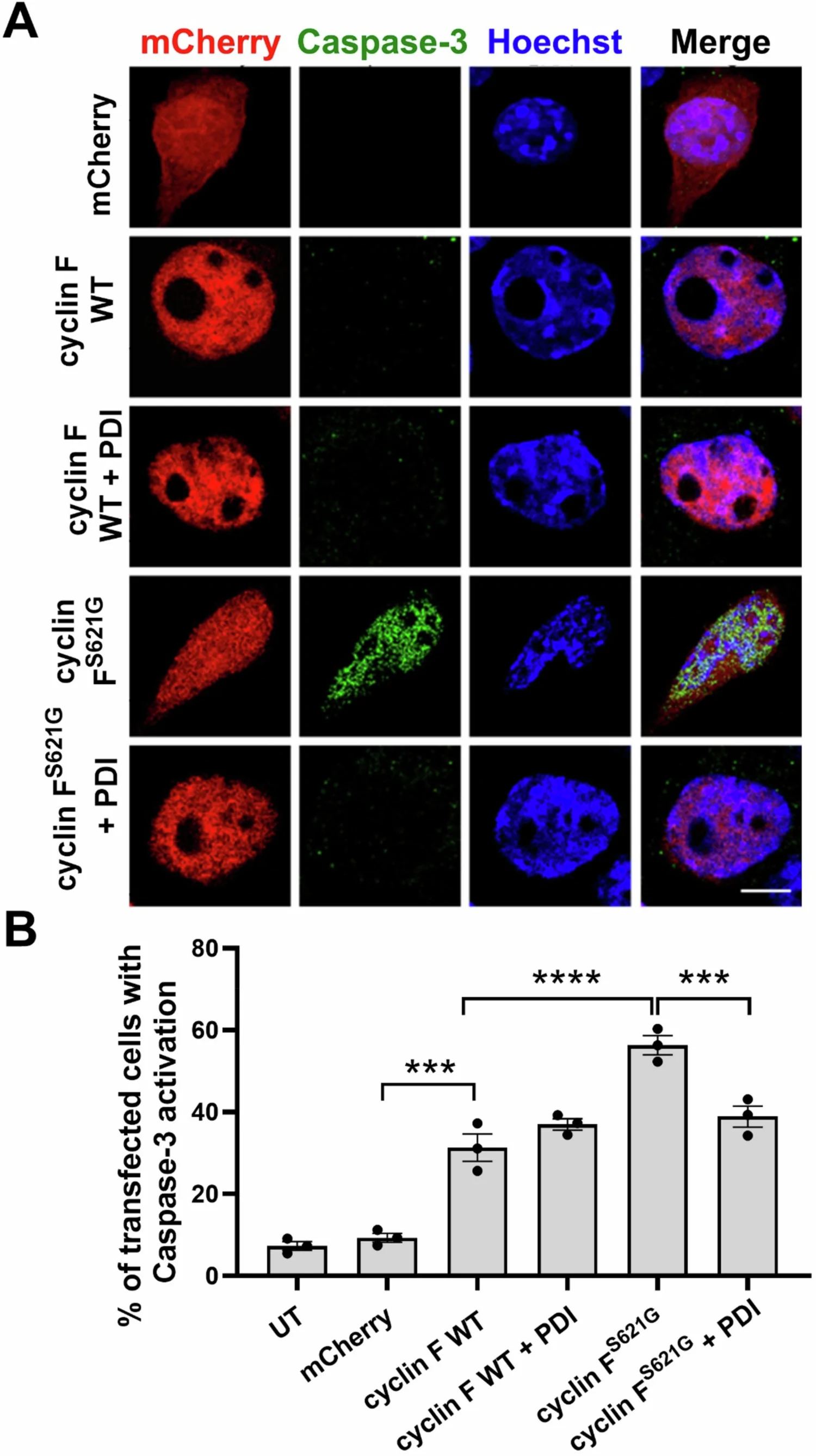
PDI overexpression inhibits the activation of Caspase-3 induced by mutant cyclin F. (A) Neuro-2a cells expressing mCherry alone (first panel), wildtype cyclin F co-expressed with either an empty vector (second panel; cyclin F WT) or PDI-V5 (third panel), or mutant cyclin F^S621G^ co-expressed with either an empty vector (fourth panel) or PDI-V5 (fifth panel). Scale bar = 30 µm. (B) Quantification of the proportion of cells with caspase-3 activation from the images in A). Significantly more mutant cyclin F^S621G^ expressing cells display caspase-3 activation compared to wildtype cyclin F cells (****p < 0.0001). The proportion of cells with caspase-3 activation was significantly reduced when PDI was co-expressed with mutant cyclin F^S621G^ (***p < 0.001). There was no significant difference in the proportion of cells displaying caspase-3 activation between populations expressing wildtype cyclin F and either empty vector pcDNA3.1 or PDI. *N* = 3, Mean ± SEM, ***p < 0.001, ****p < 0.0001.

### PDI prevents cyclin F-induced motor impairment in vivo in zebrafish

We next examined whether PDI is protective in a previously described zebrafish larvae ALS model expressing mutant cyclin F^S621G^ [15] that displays impaired motor performance [15]. mRNA encoding PDI tagged with mKate [27] (or control mKate only) was co-injected with mRNA encoding either cyclin F wildtype or mutant^S621G^, into 1–4 cell stage zebrafish embryos. Motor performance was examined using a ZebraBox automated tracking system [15,27] at 48 h post fertilisation (hpf), which automatically tracks the distance swam in response to a flash of light (photomotor response). Zebrafish larvae expressing cyclin F^S621G^ travelled significantly shorter distances compared to non-injected controls and those expressing wildtype cyclin F (Figure 7, *p< 0.05), as previously described [15]. However, zebrafish larvae co-expressing PDI-mKate2 with mutant cyclin F^S621G^ travelled significantly further distances compared to those co-expressing cyclin F^S621G^ with mKate2 only (Figure 7, *p< 0.05). Hence, PDI protects against mutant cyclin F^S621G^-mediated motor impairment in zebrafish embryos. Together, these results therefore demonstrate that PDI is protective *in vivo* against mutant cyclin F^S621G^-induced motor impairment in a zebrafish model.

**Figure 7.**
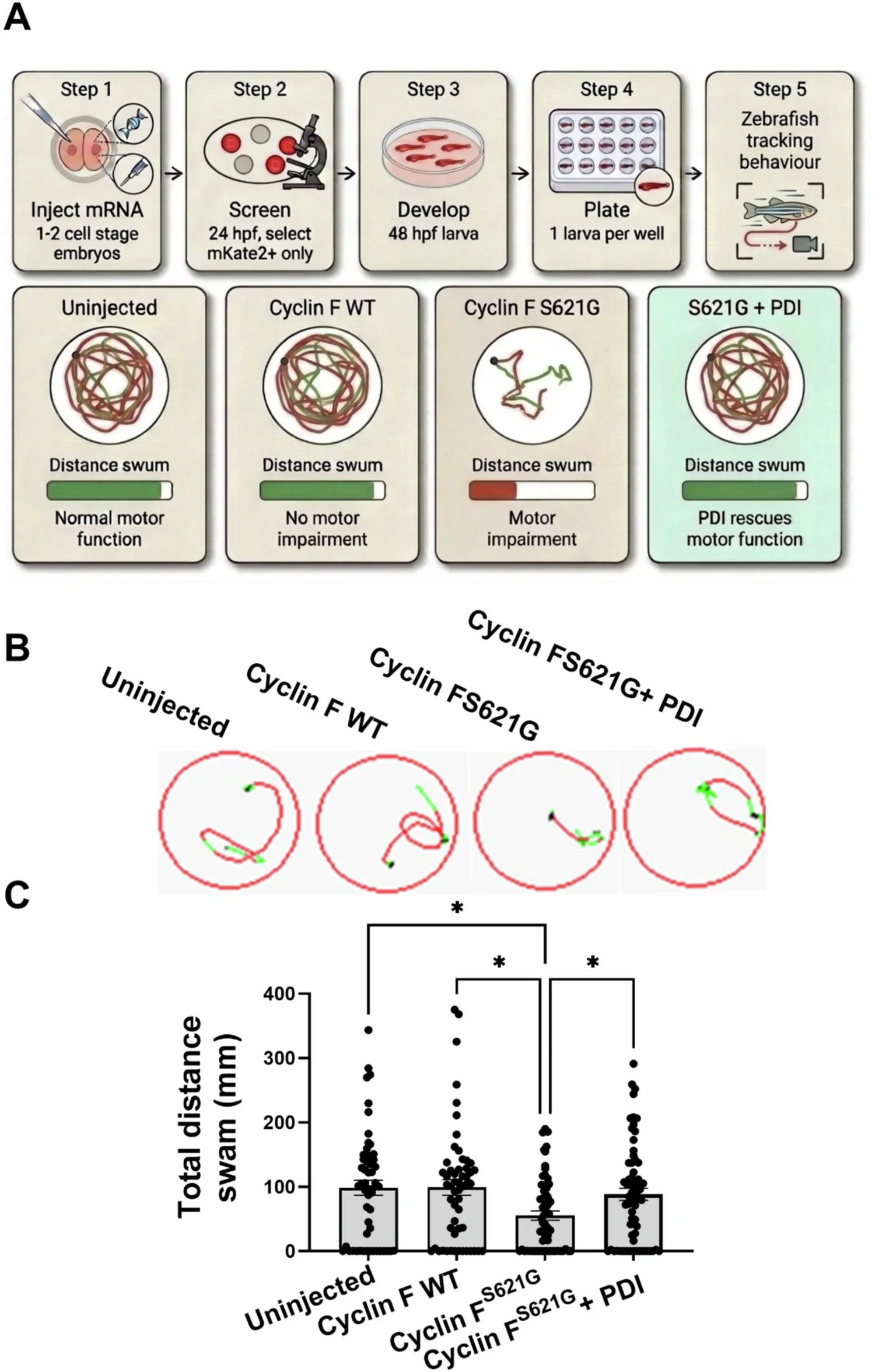
PDI prevents mutant cyclin F^S621G^-induced motor impairment in zebrafish larvae. (A) Schematic diagram of the motor impairment protocol and data interpretation. Human cyclin F WT or cyclin F^S621G^ mRNA was co-injected with mKate2 mRNA or PDI-mKate2 into 1–4-cell-stage zebrafish embryos (Step 1). At 24 hpf, the embryos were screened on a fluorescent stereomicroscope, and only mKate2-positive (successfully injected) embryos were retained for analysis (Step 2). The embryos were allowed to develop until 48 hpf (Step 3), then they were distributed individually into 96-well plates (Step 4). After a 30-minute dark acclimation, the larvae were exposed to three 1-second flashes of light at 1-minute intervals using a Zebrabox automated tracking device, and the total distance swum over the 3-minute recording period was measured (Step 5). The lower panels summarise the motor phenotype of each of the four experimental conditions. Schematic in (A) created with FigureLabs (License number: FL-PUB-20260819-BIBA0N). (B) Representative images illustrate the trajectory of movement of individual 48 hpf zebrafish larvae. The red lines indicate fast movement (>8 mm/sec), the green lines represent slow movement (3–8 mm/sec), and the black lines show inactivity (<3 mm/sec). (C) Un-injected zebrafish larvae and larvae expressing wildtype cyclin F swam significantly further than those expressing mutant cyclin F^S621G^ (*n* > 55 per group, *p < 0.05). Co-expression of PDI-mKate2 with cyclin F^S621G^ significantly attenuated the motor impairment induced by cyclin F^S621G^ compared to the mKate only group (*n* > 55 per group, *p < 0.05). Mixed-effects analysis followed by Tukey's multiple comparisons post-hoc test. All values represent mean ± SEM, *p < 0.05.

## Discussion

This study demonstrates that PDI is protective against key phenotypes associated with ALS/FTD-mutant cyclin F^S621G^, including motor impairment *in vivo*. Several mechanisms of pathogenesis are associated with mutant cyclin F^S621G^ , which were examined in this study. Mutant cyclin F^S621G^ mislocalises from the nucleus to the cytoplasm, similar to mutant TDP-43 and FUS, where it induces dysfunction to the proteasome and apoptosis [13]. The findings of this study revealed that PDI prevents cyclin F mis-localisation from the nucleus to the cytoplasm, proteasome dysfunction, ER stress, and apoptosis. It also restores motor function in a cyclin F^S621G^ zebrafish model. PDI is upregulated in ALS patients, but it undergoes post-translational modification and is S-nitrosylated, therefore losing its normal protective function in ALS [44]. It is possible then, that over-expressing PDI may compensate for the loss of endogenous PDI activity, and thus, is able to overcome these cellular defects induced by mutant cyclin F. Hence, these data imply that therapeutics based on PDI may have potential for ALS/FTD cases associated with cyclin F mutations.

PDI and related family member, ERp57, show broad protective activity against multiple proteins and a wide range of phenotypes associated with ALS. This includes cellular and/or zebrafish models based on SOD1, TDP-43, and FUS, and the widely used SOD1^G93A^ mouse model, where it improves neuromuscular connectivity [27,28,31,49,50]. The overexpression of PDI and ERp57 enhances neurite outgrowth in motor neuronal cells, primary neurons, and human motor neurons produced from differentiation of embryonic stem cells [33]. Moreover, ERp57 is protective at the synapse [33]. Conditional ERp57 knockout mice display abnormal synapses, reduced muscle innervation, aberrant skeletal muscle fibres and dysfunctional neuromuscular connectivity, resulting in a significant decline in motor performance, a reduction in body mass, and premature death [33]. Hence, these data suggest that PDI family members have novel roles in motor control and neuromuscular connectivity. The findings of this study are consistent with these observations. In the mutant cyclin F^S621G^ zebrafish model displaying impaired swimming ability, the co-expression of PDI attenuated this motor impairment significantly. Hence, these findings reveal that PDI is protective against motor phenotypes in a mutant cyclin F^S621G^ zebrafish model.

PDI was found to inhibit mutant cyclin F^S621G^ localisation in the cytoplasm, despite being a normally ER-resident protein. Interestingly, however, PDI has been shown to redistribute away from the ER as punctate vesicular structures by reticulon proteins, and in this location, PDI was observed to have higher enzymatic activity [51]. Moreover, the knockdown of reticulon 4 A and 4B accelerates axonal degeneration and early death of transgenic SOD1^G93A^ mice, suggesting that the redistribution of PDI away from the ER modulates disease progression [52]. PDI also relocates to the nucleus, where it is protective against DNA damage [25]. In fact, PDI has been detected in the cytoplasm, nucleus, and extracellular space, although the mechanisms by which PDI leaves the ER in these instances are poorly understood [53]. These studies suggest that PDI plays a protective role outside of the ER and that the location of PDI is relevant to disease in ALS. Therefore, it may be possible that PDI exhibits its protective function against mutant cyclin F in the cytoplasm in ALS, although further studies are required to confirm this. As a chaperone, it is possible that PDI interacts directly with misfolded mutant cyclin F, although this interaction was not assessed here. An interaction between PDI and mutant cyclin F could mitigate the phenotypes assessed in this study. Alternatively, PDI may act indirectly through broader effects on ER-associated protein quality control, or redox and proteostasis regulation. Future studies are required to define the precise mechanism of action of PDI and whether this involves its direct interaction with mutant cyclin F.

ER stress is implicated in the pathogenesis of ALS [16,54]. Two markers of the UPR were used to investigate the activity of PDI against the previously described mutant cyclin F-induced ER stress [16]. XBP-1 is a marker of the early phase of the UPR, and CHOP activation is an indicator of the late phase of the UPR [55,56], whereby the UPR switches from its protective to pro-apoptotic phases. Hence, this study demonstrated a protective role for PDI during both the early and late phases of the UPR induced by the mutant cyclin F^S621G^. We previously showed that cyclin F^S621G^ induces ER stress by inhibiting ER-Golgi transport [16]. Previous studies from our group and others have proposed that mutant SOD1 induces ER stress from the cytoplasm [57,58] and that mutant TDP-43 triggers ER stress from the cytoplasmic face of the ER, also by inhibiting ER-Golgi transport [59]. Hence, it remains unclear whether mutant cyclin F induces ER stress from either the ER or cytoplasm, or both.

It should be noted that consistent with previous observations [16] wildtype cyclin F induced low levels of ER stress compared to mCherry only, and importantly, PDI had no effect on the phenotypes induced by wildtype cyclin F except UPS dysfunction. However, there was a statistically significant difference in phenotypes detected between cells expressing wildtype cyclin F and mutant cyclin F^S621G^. The ER has a finite capacity for protein synthesis and folding, and even correctly folded wildtype proteins can exceed the ER processing capacity. Hence, it is possible that in wildtype cyclin F-expressing cells, ER stress may be induced by high levels of protein overexpression in Neuro2a cells. In contrast, in cells expressing misfolded, mutant cyclin F, there is an additional burden of misfolded cyclin F protein. PDI is a chaperone that prevents protein misfolding, and it is well established that it translocates to the cytoplasm during ER stress [60]. Hence, it is possible that PDI inhibits the misfolding of mutant cyclin F and thus the additional ER stress induced by protein misfolding. However, PDI may be unable to prevent the ER stress induced simply by the overexpression of either wildtype or mutant cyclin F. Consistent with this hypothesis, in each experiment, we observed that in PDI-expressing mutant cyclin F cells, the levels of ER stress, UPS dysfunction or apoptosis were reduced to a level comparable to wildtype cyclin F. Hence, it is possible that there are two mechanisms inducing ER stress in this study, high levels of protein overexpression and protein misfolding, and that PDI prevents the latter, but not the former, process. However, we cannot rule out the possibility that there are also additional mechanisms responsible for inducing ER stress in the case of wildtype cyclin F.

Alternatively, PDI could promote protein degradation in order to reduce protein load by facilitating ERAD [61] because mutant cyclin F^S621G^ impedes this process [16]. Mutant cyclin F abnormally increases the ubiquitination of TDP-43 and its transport to the proteasome, and thus may be accountable for wider changes in protein homoeostasis [4]. Therefore, it may be possible that mutant cyclin F accelerates the presence of abnormal misfolded proteins, such as TDP-43, and the accumulation of protein aggregates, and that PDI plays a protective role in preventing these defects, as has been previously reported for SOD1.

The finding that wildtype cyclin F induces some defects is consistent with previous observations that elevated cyclin F expression can perturb cellular homoeostasis, likely through increased engagement of the ubiquitin-proteasome and ER-associated protein quality control pathways [16]. In addition, previous studies have shown that the overexpression of wildtype cyclin F can still influence ubiquitination dynamics and protein homoeostasis due to its central role as an SCF E3 ubiquitin ligase component [4]. However, these effects were consistently less pronounced than those induced by the ALS/FTD-linked cyclin F^S621G^, supporting the view that the S621G mutation confers an enhanced pathogenic effect beyond cyclin F overexpression alone.

PDI is upregulated during the induction of the UPR [18] and as an oxidoreductase, it functions by refolding misfolded proteins into their native conformation [62], preventing the formation of mutant SOD1, TDP-43, and FUS inclusions [27,28]. Neither misfolded or aggregated cyclin F, nor visible inclusions were formed in the cells used in this study; thus, it remains unclear if PDI prevents the misfolding of cyclin F. PDI interacts with VAPB inclusions in a *Drosophila melanogaster* model of ALS [63], and mutations in VAPB have been shown to induce ER stress [64], implying that PDI has a broader role than previously recognised.

While this study provides robust evidence that PDI is protective against multiple ALS/FTD-associated phenotypes induced by mutant cyclin F, a few limitations should be noted. The cyclin F^S621G^ mutation was selected for this study, as it is the most extensively characterised *CCNF* mutation that is known to induce UPS dysfunction, ER stress and motor impairment in zebrafish [15,16]. However, other *CCNF* variants have been identified in ALS/FTD patients, and it remains to be determined whether PDI is similarly protective against these mutations. Neuro-2a cells were chosen as a well-established neuronal cell model for studying ALS-associated mechanisms. However, as an immortalised mouse neuroblastoma line, these cells do not fully recapitulate the biology of human motor neurons, so this was complemented by validation in our zebrafish model. Furthermore, a single time point of 72 h post-transfection was used for all the cellular assays, and therefore, the temporal dynamics of PDI's protective effects were not explored. Finally, although previous studies have demonstrated that the redox activity of PDI is required for its protective effects against mutant SOD1 and TDP-43, this study did not include a catalytically inactive PDI mutant to determine whether the protective effects observed here similarly require PDI's oxidoreductase activity, its chaperone function, or both.

In summary, this study is the first to demonstrate that PDI is protective against mutant cyclin F-induced mechanisms relevant to ALS pathogenesis. A hypothetical working model schematic outlining these protective activities of PDI against ALS/FTD-associated cyclin F is shown in Figure 8. The possible mechanisms involved include proteostasis dysfunction, ER-associated protein quality control, redox regulation and chaperone-like activity. Future studies will be required to determine whether reduced ER stress/UPR activation is a primary driver of protection or a downstream consequence of broader PDI-mediated effects on proteostasis. PDI is known to be broadly protective against ALS-associated proteins, implying that it is a potential therapeutic target in ALS. This study extends these previous findings and implies that therapeutics-based PDI may also lead to novel treatments for ALS/FTD cases associated with cyclin F.

**Figure 8.**
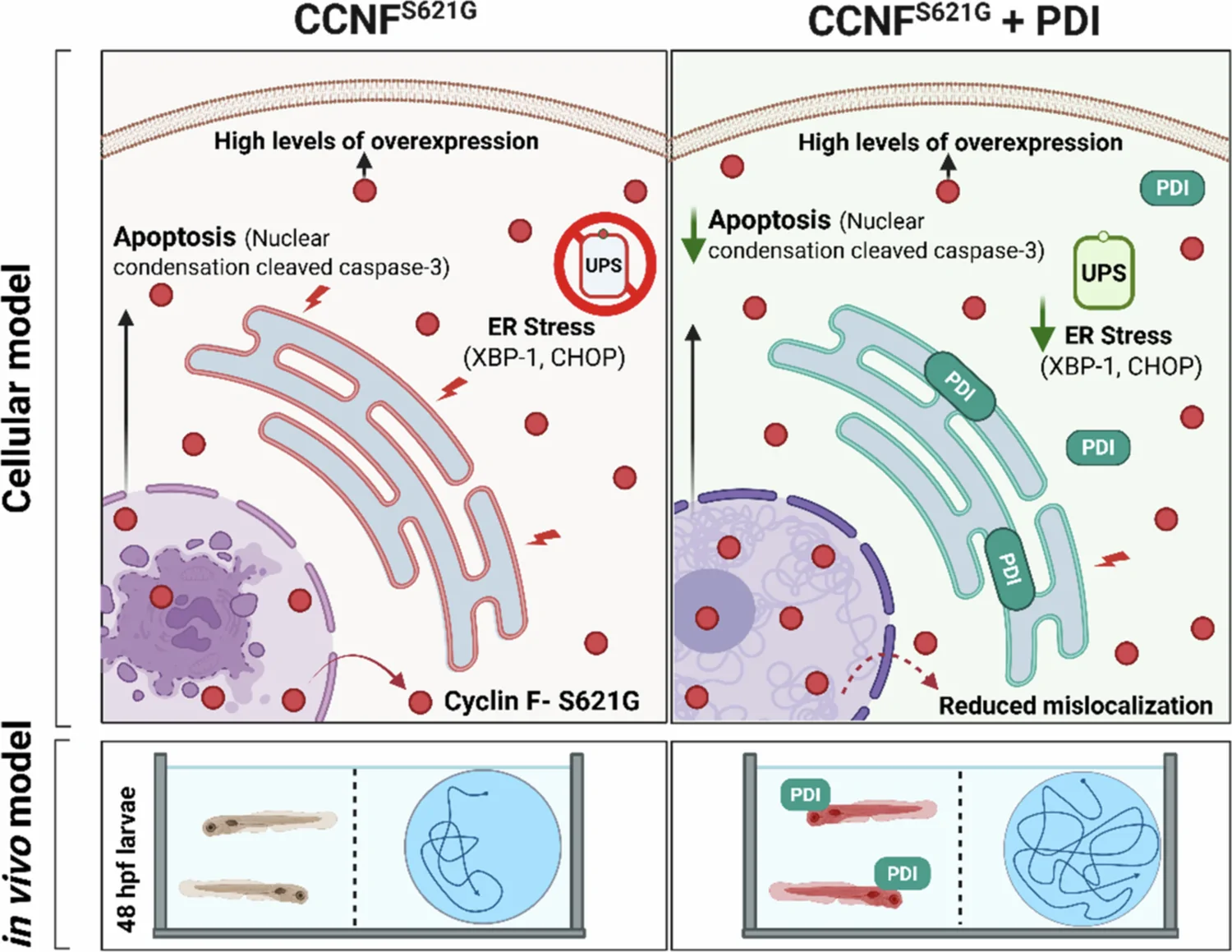
Hypothetical working model based on the findings from the current study. Mutant cyclin F^S621G^ mislocalises from the nucleus to the cytoplasm, where it induces more ER stress, UPS dysfunction, and apoptotic signalling in neuronal cells than wildtype cyclin F. These cellular phenotypes are accompanied by motor impairment in zebrafish larvae expressing mutant cyclin F^S621G^. PDI overexpression attenuates the mutant cyclin F-associated cellular phenotypes and motor impairment *in vivo*. The precise mechanism by which PDI mediates these protective effects remains to be fully determined and may involve multiple interconnected pathways, including redox/oxidoreductase activity, chaperone-like activity, ER-associated protein quality control, and broader proteostasis regulation. Low levels of ER stress are induced by the overexpression of wildtype cyclin F, but PDI is unable to resolve this phenomenon. Figure created with BioRender.com; license number: TW2A3EPQ2B.

## Supplementary Material

Supplementary Figure.docx Supplementary Figure.docx

Shadfar et al ARRIVE.pdf Shadfar et al ARRIVE.pdf

## Data Availability

The data that support the findings of this study are available from the corresponding author upon reasonable request.
