# Supplementary material for: Protein disulphide isomerase protects against ALS/FTD phenotypes associated with cyclin F in zebrafish and cellular models
Source: Redox Rep. 2026 Oct 18;31(1):2732775. doi: 10.1080/13510002.2026.2732775 (PMC13686195; doi:10.1080/13510002.2026.2732775)
Supplement: Supplementary file 1 [file YRER_A_2732775_SM2959.pdf]

NOTE: Please save this file locally before filling in the table, DO NOT work on the file within your internet browser as changes will not be saved. Adobe Acrobat Reader (available free here) is recommended for completion.

The ARRIVE guidelines 2.0: author checklist

The ARRIVE Essential 10

These items are the basic minimum to include in a manuscript. Without this information, readers and reviewers cannot assess the reliability of the findings.

Item		Recommendation	Section/line number, or reason for not reporting
**Study design**	1	For each experiment, provide brief details of study design including:	
		a. The groups being compared, including control groups. If no control group has been used, the rationale should be stated.	p.5-11(in vivo groups: uninjected; cyclin F WT + mKate2; cyclin FS621G + mKate2; cyclin FS621G + PDI-mKate2).
		b. The experimental unit (e.g. a single animal, litter, or cage of animals).	p.11, line 246(the experimental unit was a single zebrafish larva, one larva per well). Cellular experimental unit = independent biological replicate, N = 3
**Sample size**	2	a. Specify the exact number of experimental units allocated to each group, and the total number in each experiment. Also indicate the total number of animals used.	p.11, lines 246-252(uninjected n=55; cyclin F WT n=55; cyclin FS621G n=66; cyclin FS621G + PDI n=66; total = 242 larvae, 3 biological replicates). Also Figure 7 legend, p.33, lines 540-559
		b. Explain how the sample size was decided. Provide details of any *a priori* sample size calculation, if done.	p.11, lines 250-251 (no a priori sample-size calculation; group sizes based on previous zebrafish photomotor ALS/FTD studies).
**Inclusion and exclusion criteria**	3	a. Describe any criteria used for including and excluding animals (or experimental units) during the experiment, and data points during the analysis. Specify if these criteria were established *a priori*. If no criteria were set, state this explicitly.	p.10, lines 223-230 (mKate2-positive and morphologically normal embryos included; abnormal or non-expressing embryos excluded; criteria set a priori).
		b. For each experimental group, report any animals, experimental units or data points not included in the analysis and explain why. If there were no exclusions, state so.	p.10, lines 225-208 (embryos failing screening excluded before analysis, accounting for the differing final group sizes).
		c. For each analysis, report the exact value of *n* in each experimental group.	p.11, lines 246-252 (uninjected n=55; cyclin F WT n=55; cyclin FS621G n=66; cyclin FS621G + PDI n=66). Figure 7 legend, p.31, lines 463-466.
**Randomisation**	4	a. State whether randomisation was used to allocate experimental units to control and treatment groups. If done, provide the method used to generate the randomisation sequence.	p.9, lines 218-223(following spawning, fertilised embryos were pooled and randomly allocated to the experimental groups prior to microinjection).
		b. Describe the strategy used to minimise potential confounders such as the order of treatments and measurements, or animal/cage location. If confounders were not controlled, state this explicitly.	p.10, lines 229-231 (all groups raised in equal numbers under identical conditions: E3 medium, darkness, 28C). p.10, lines 235-236 (uniform 30-minute dark acclimatisation before tracking).
**Blinding**	5	Describe who was aware of the group allocation at the different stages of the experiment (during the allocation, the conduct of the experiment, the outcome assessment, and the data analysis).	p.10, lines 225-228 (allocation coded by an independent researcher, Kristy Yuan; investigators performing tracking, outcome assessment and analysis blinded until analysis complete). Cellular work also blinded: p.8, lines 176-177.
**Outcome measures**	6	a. Clearly define all outcome measures assessed (e.g. cell death, molecular markers, or behavioural changes).	In vivo outcome (total distance swum in the photomotor response): p.10 lines 238- 239; Figure 7, p.34. Cellular outcomes (mislocalisation; XBP-1; CHOP; GFPu; apoptotic nuclei; cleaved caspase-3) defined
		b. For hypothesis-testing studies, specify the primary outcome measure, i.e. the outcome measure that was used to determine the sample size.	Primary in vivo outcome = total distance swum (Results, p.31, lines 518-532; Figure 7).
**Statistical methods**	7	a. Provide details of the statistical methods used for each analysis, including software used.	p.11, lines 257-267 (one-way ANOVA with Tukey post hoc; GraphPad Prism 10.2; data as mean +/- SEM). Zebrafish data: Figure 7 legend, p.33, lines 554-556 (mixed-effects analysis with Tukey multiple comparisons).
		b. Describe any methods used to assess whether the data met the assumptions of the statistical approach, and what was done if the assumptions were not met.	p11, lines 257-267: The Statistical Analysis section states that data normality was assessed using the Shapiro-Wilk test, and non-parametric tests were used when normality assumptions were not met
**Experimental animals**	8	a. Provide species-appropriate details of the animals used, including species, strain and substrain, sex, age or developmental stage, and, if relevant, weight.	p.9 lines 215- 217(zebrafish, Danio rerio; transgenic Tg(-7933mnx1:TagBFP)mq on a TAB-WT background). Developmental stage: p.31, lines 528-531(1-4 cell injection; 24 hpf screen; 48 hpf assay). Sex not
		b. Provide further relevant information on the provenance of animals, health/immune status, genetic modification status, genotype, and any previous procedures.	p.9, lines 211-217(bred in-house from the established Macquarie University colony; stable transgenic reporter line, standard housing).
**Experimental procedures**	9	For each experimental group, including controls, describe the procedures in enough detail to allow others to replicate them, including:	
		a. What was done, how it was done and what was used.	p.10, l(mRNA co-injection, 0.905 nl droplets, Picospritzer II; screening on Leica M165FC stereomicroscope). p.10 lines 235 (photomotor tracking on a Zebrabox).
		b. When and how often.	Injection at 1-4 cell stage (p.9); screening at 24 hpf (p.10, lines 182-184); tracking at 48 hpf, three 1-second light flashes at 1-minute intervals over 3 minutes
		c. Where (including detail of any acclimatisation periods).	p.10, line 235(larvae raised in E3 medium, darkness, 28C). p.10 (placed individually in 96-well plates; 30-minute dark acclimatisation inside the Zebrabox before
		d. Why (provide rationale for procedures).	Results, p.31, lines 524-538 (cyclin FS621G zebrafish photomotor model selected as a validated in vivo ALS model with a quantifiable motor phenotype).
**Results**	10	For each experiment conducted, including independent replications, report:	
		a. Summary/descriptive statistics for each experimental group, with a measure of variability where applicable (e.g. mean and SD, or median and range).	Data presented as mean +/- SEM for each group throughout (Figures 1-7 legends). Zebrafish: Figure 7 legend, p.33, (n=55-66 per group). Cellular: N = 3, at least 100 cells per treatment.
		b. If applicable, the effect size with a confidence interval.	Effect sizes and confidence intervals were not calculated; comparisons reported as mean +/- SEM with p-values.

The Recommended Set

These items complement the Essential 10 and add important context to the study. Reporting the items in both sets represents best practice.

Item		Recommendation	Section/line number, or reason for not reporting
**Abstract**	11	Provide an accurate summary of the research objectives, animal species, strain and sex, key methods, principal findings, and study conclusions.	p.2, lines 29-52
**Background**	12	a. Include sufficient scientific background to understand the rationale and context for the study, and explain the experimental approach.	p.3-5, lines 56-103
		b. Explain how the animal species and model used address the scientific objectives and, where appropriate, the relevance to human biology.	p.3, lines 74-76<br>p.34, lines 584-588
**Objectives**	13	Clearly describe the research question, research objectives and, where appropriate, specific hypotheses being tested.	p.2, lines 37-38 p.4, lines 97-103
**Ethical statement**	14	Provide the name of the ethical review committee or equivalent that has approved the use of animals in this study, and any relevant licence or protocol numbers (if applicable). If ethical approval was not sought or granted, provide a justification.	p.9, lines 212-215; p.40-41, lines 721-724 .
**Housing and husbandry**	15	Provide details of housing and husbandry conditions, including any environmental enrichment.	p.9, lines 212-217 .
**Animal care and monitoring**	16	a. Describe any interventions or steps taken in the experimental protocols to reduce pain, suffering and distress.	p.11, lines 253-256 (humane euthanasia by rapid immersion in dilute sodium hypochlorite per AVMA Guidelines 2020; larvae assessed only to 48 hpf, euthanised before 5 dpf).
		b. Report any expected or unexpected adverse events.	No expected or unexpected adverse events were reported. Embryos with developmental abnormalities were identified and excluded at 24 hpf screening (p.8, lines 183-186).
		c. Describe the humane endpoints established for the study, the signs that were monitored and the frequency of monitoring. If the study did not have humane endpoints, state this.	No disease-progression humane endpoints were set; larvae were euthanised before 5 dpf (p.11 lines 225-227).
**Interpretation/ scientific implications**	17	a. Interpret the results, taking into account the study objectives and hypotheses, current theory and other relevant studies in the literature.	p.33-39, Discussion
		b. Comment on the study limitations including potential sources of bias, limitations of the animal model, and imprecision associated with the results.	Discussion, p.38, lines 670-685
**Generalisability/ translation**	18	Comment on whether, and how, the findings of this study are likely to generalise to other species or experimental conditions, including any relevance to human biology (where appropriate).	p.38-39, lines 686-696;
**Protocol registration**	19	Provide a statement indicating whether a protocol (including the research question, key design features, and analysis plan) was prepared before the study, and if and where this protocol was registered.	Not reported. No protocol was pre-registered for this study.
**Data access**	20	Provide a statement describing if and where study data are available.	p.41, lines 725-726
**Declaration of interests**	21	a. Declare any potential conflicts of interest, including financial and non-financial. If none exist, this should be stated.	p.41, lines 727-728
		b. List all funding sources (including grant identifier) and the role of the funder(s) in the design, analysis and reporting of the study.	p.40, lines 718-720
