# Supplementary material for: Protein disulphide isomerase protects against ALS/FTD phenotypes associated with cyclin F in zebrafish and cellular models
Source: Redox Rep. 2026 Oct 18;31(1):2732775. doi: 10.1080/13510002.2026.2732775 (PMC13686195; doi:10.1080/13510002.2026.2732775)
Supplement: Supplementary file 2 [file YRER_A_2732775_SM2958.docx]

**Supplementary Figure 1**

**Supplementary figure 1: Expression of PDI-V5 and Cyclin F-mCherry proteins in Neuro-2A cells.** Representative Western blots from Neuro-2a cell lysates expressing mCherry vector alone, wildtype cyclin F, wildtype cyclin F with PDI-V5, mutant cyclin F^S621G^, or mutant cyclin F^S621G^ with PDI-V5. β-actin was used as a loading control.
